# Prognostic Value of International Normalized Ratio and Thrombocytopenia in Early Risk Stratification of Septic Patients

**DOI:** 10.3390/biomedicines14040839

**Published:** 2026-04-07

**Authors:** Sofía Tejada, Andrés Giglio, Maria Aranda, Antonia Socias, Alberto del Castillo, Joana Mena, Sara Franco, Maria Ortega, Yasmina Nieto, Marcio Borges-Sa

**Affiliations:** 1 Multidisciplinary Sepsis Group, Health Research Institute of the Balearic Islands (IdISBa), 07120 Palma de Mallorca, Spain; sofia.tejada@idisba.es (S.T.); maranda@hsll.es (M.A.); asocias@hsll.es (A.S.); adelcastillo@hsll.es (A.d.C.); jmena@hsll.es (J.M.); sarafrancoserrano@gmail.com (S.F.); mery_6666@hotmail.com (M.O.); fmotoso@msn.com (Y.N.); 2 Fundación Código Sepsis, 46003 Valencia, Spain; 3 Critical Care Department, Clinica Las Condes Hospital, Santiago 7591046, Chile; 4 Critical Care Department, Finis Terrae University, Santiago 7640471, Chile; 5 Multidisciplinary Sepsis Unit, Intensive Care Unit, Son Llàtzer Hospital, 07198 Palma de Mallorca, Spain; 6 Faculty of Medicine, Balearic Islands University (UIB), 07122 Palma de Mallorca, Spain

**Keywords:** sepsis, coagulopathy, INR, thrombocytopenia, prognosis, sepsis-induced coagulopathy

## Abstract

**Background/Objective**: Coagulopathy is a hallmark of sepsis, associated with poor outcomes. Although platelet count has commonly been used for risk stratification, its prognostic value has remained limited. This study compared the ability of the International Normalized Ratio (INR) and platelet count to predict in-hospital mortality in septic patients; **Methods**: A retrospective study was conducted including adult patients diagnosed with sepsis and admitted to Hospital Universitario Son Llàtzer (Spain) between 2006 and 2022. The INR and platelet count at diagnosis were categorized using clinical thresholds. The primary outcome was in-hospital mortality. **Results**: Among 6433 patients (60.6% females), mortality was 8.8%. Mortality increased from 6.3% (INR ≤ 1.2) to 20.2% (INR 2.0–3.0), slightly decreasing at INR > 3.0 (10.8%). The platelet count showed a weaker association, with the highest mortality observed at <50 × 10^9^/L (24.6%). The combined markers identified a high-risk subgroup with 50% mortality (INR > 3.0 and platelet count < 50 × 10^9^/L). In the full cohort, multivariable analysis confirmed the INR as an independent predictor of mortality (OR 2.183, *p* = 0.0002), whereas the platelet count was not significant. The model including the INR achieved an AUC 0.746, while adding the platelet count did not improve performance. **Conclusions**: the INR at diagnosis was a strong and independent predictor of in-hospital mortality, outperforming the platelet count. These findings could support the consideration of the INR in early risk stratification frameworks and highlight the need for prospective validation before integration into sepsis guidelines.

## 1. Introduction

Sepsis is a life-threatening condition caused by a dysregulated host response to infection and frequently resulting in organ dysfunction [1]. It remains one of the leading causes of in-hospital mortality worldwide [2]. Coagulation abnormalities play a central role in its pathophysiological [3,4], ranging from subclinical activation of the coagulation cascade to overt disseminated intravascular coagulation (DIC), with direct implications for organ failure and survival [5,6].

The International Normalized Ratio (INR) and platelet count are widely available coagulation parameters used in routine clinical practice. Platelet counts play an essential role in hemostasis and immunity, and reduced platelet counts have been associated with poor outcomes in several clinical settings [7,8,9]. Thrombocytopenia affects up to 50% of septic patients [8,10]. It may arise from multiple mechanisms (e.g., consumption, splenic sequestration, or immune-mediated destruction) which limits its specificity as a prognostic biomarker [7,8,9].

The INR reflects the activity of the extrinsic coagulation pathway and may serve as a direct and sensitive indicator of early hemostatic dysfunction [11]. Elevated values may arise from hepatic dysfunction, consumption of coagulation factors, or systemic inflammation, all of which are common features of sepsis. Although the INR has traditionally been used to monitor anticoagulation therapy, its prognostic relevance in sepsis has become increasingly evident.

The platelet count is included in the Sequential Organ Failure Assessment (SOFA) score, whereas the INR is not incorporated into Sepsis-3. Despite this exclusion, the INR remains biological relevant because it reflects the extrinsic pathway activity and hepatic synthetic function [12,13]. Emerging evidence has indicated that the INR may offer complementary prognostic information [6]. Its potential role in early risk stratification should be interpreted cautiously and requires prospective validation before clinical use [14].

Previous studies have reported an association between an elevated INR and increased mortality in sepsis [15,16,17]; however, most of them have focused on intensive care unit (ICU) populations or relied on composite scores rather than assessing the INR as an independent predictor. Furthermore, a direct comparison between the INR and platelet count as competing prognostic markers has not been rigorously examined using flexible non-linear modeling.

This study aims to evaluate and compare the prognostic value of the INR and platelet count for predicting in-hospital mortality in a large, heterogeneous, hospital-wide sepsis cohort.

## 2. Materials and Methods

### 2.1. Study Design and Population

A retrospective observational cohort study was conducted at Hospital Universitario Son Llàtzer, a 350-bed tertiary care university hospital in Palma de Mallorca, Spain. All adults (≥18 years) with sepsis and septic shock identified through a modified Sepsis-2 protocol requiring organ dysfunction (equivalent to severe sepsis) were included between January 2006 and December 2022 [18]. The institutional sepsis code, implemented in 2006, captured activations from the emergency department, general wards, and the ICU [18]. Patients without INR or platelet count measurements at the time of sepsis-code activation were excluded. The details of the sepsis protocol activation system are provided in Supplemental Section S1.

The primary outcome was in-hospital mortality.

### 2.2. Data Collection

Clinical data were extracted from electronic medical records. The demographic variables were recorded together with the care setting (emergency department, hospital ward, ICU).

Laboratory parameters reflecting inflammatory status and organ function were collected, including the white blood cell (WBC) count, C-reactive protein (CRP), and procalcitonin (PCT). Additional biochemical markers (bilirubin, creatinine, and cholesterol) were used to assess hepatic, renal, and metabolic functions. Clinical signs of organ dysfunction (altered mental status, oliguria, and hypoxemia) were also recorded at the time of sepsis diagnosis.

Coagulation status was assessed using platelet counts, fibrinogen levels, and an INR. Because the analysis focused on the INR at the time of diagnosis, clinical validators evaluated whether each INR elevation reflected acute sepsis-associated coagulopathy or baseline conditions such as chronic liver disease or anticoagulant therapy (e.g., cirrhosis or anticoagulated patients).

### 2.3. Definitions

The INR and platelet count were measured at the time of sepsis-code activation (i.e., at clinical diagnosis) before the initiation of specific treatments.

Organ dysfunction was quantified using a non-coagulator dysfunction score, defined as the sum of seven binary criteria: hypoxemia, oliguria, altered consciousness, elevated creatinine, hyperbilirubinemia, and hyperlactatemia. The coagulation component was excluded to avoid double-counting when coagulation markers were used as predictors.

Thrombocytopenia was defined as a platelet count < 150 × 10^9^/L and classified into the following categories [19]: severe (<50 × 10^9^/L); moderate (50–99 × 10^9^/L); mild (100–149 × 10^9^/L); normal (150–399 × 10^9^/L); elevated (400–590 × 10^9^/L); and very high (≥600 × 10^9^/L).

Coagulopathy was assessed using the INR. Values >1.2 were considered indicative of altered coagulation. For risk stratification, the INR was categorized into five predefined ranges based on the previous literature [20]: normal (≤1.2); mildly elevated (1.2–1.5); moderately elevated (1.5–2.0); high (2.0–3.0); and very high (>3.0).

Pre-existing coagulopathy was identified at the time of sepsis-code activation. SIC has been defined elsewhere [6,21]. In this study, SIC was assigned when the treating clinician attributed the coagulation abnormality to sepsis, excluding patients with pre-existing coagulopathy and/or thrombocytopenia, therapeutic anticoagulation, or known chronic liver disease. Within the SIC cohort (N = 1421), four mutually exclusive coagulation phenotypes were defined:

- Elevated INR only: INR ≥ 1.5 and platelet count ≥ 150 × 10^9^/L.
- Thrombocytopenia only: INR < 1.5 and platelet count < 150 × 10^9^/L.
- Both altered-DIC pattern: INR ≥ 1.5 and platelet count < 150 × 10^9^/L.
- Mild/subthreshold alterations: INR of 1.2–1.5.

To minimize classification bias, each episode with an elevated INR was reviewed at the time of sepsis-code activation. The elevation was attributed to sepsis only when no chronic explanation (e.g., anticoagulants or chronic liver disease) was identified. Cases with an uncertain origin were classified as non-sepsis-related coagulopathy. This process enabled a strong distinction between sepsis-induced hemostatic dysfunction and chronic disorders, ensuring appropriate interpretation of coagulation parameters.

### 2.4. Statistical Analysis

All analyses were performed using R Studio 4.3.1 and Python 3.12, including stats models (logistic regression), patsy (spline basis), and scikit-learn (AUROC). A *p*-value < 0.05 was considered statistically significant.

Continuous variables were presented as means with standard deviations (SDs) or medians with interquartile ranges [IQRs]. Group comparisons were performed using the Mann–Whitney U test or Kruskal–Wallis’s test. Categorical variables were reported as frequencies (%) and compared using Fisher’s exact test or chi-square test.

Logistic regression was used to assess the prognostic value of the INR, including both linear and quadratic terms. Odds ratios (ORs) with 95% confidence intervals (CIs) were calculated for associations between coagulation parameters and clinical outcomes. Multivariable models were adjusted for age and number of organ dysfunctions. Additional models included baseline coagulopathy variable (yes/no), hyperlactatemia (when available), and the infection source and bacteremia (dummy-coded when missing). The length of stay was excluded as a post-outcome variable to avoid collider bias.

A sensitivity analysis excluded patients with pre-existing causes of coagulopathy (i.e., chronic liver disease or anticoagulant therapy) based on clinical assessment at diagnosis. This allowed the evaluation of whether the prognostic value of the INR was specific to SIC.

To assess non-linearity, cubic B-spline regression models (five degrees of freedom) were fitted for the INR and platelet count, adjusted for age and number of organ dysfunctions. The 95% confidence intervals (CIs) were estimated by bootstrap resampling (300 iterations). Model discrimination was evaluated using AUROC and compared across the linear and spline models.

The combined INR and platelet categories were created to assess mortality risk across coagulation profiles. ORs with 95% CIs were calculated using an INR ≤ 1.2 and a platelet count of 150–400 × 10^9^/L as the reference group. The Haldane correction (adding 0.5 to each cell) was applied when zero events occurred.

Subgroup analyses were stratified by age, sex, care setting, number of organ dysfunctions, and coagulopathy status. Associations between the INR and the outcomes were evaluated using logistic regression. The linear or quadratic models were selected based on the significance of the quadratic term (*p* < 0.10). Forest plots were used to display subgroup effects, with logarithmic scale to account for wide confidence intervals.

For descriptive analyses, the INR and platelet count were categorized using pre-specified clinical thresholds. The INR was modeled using three functional forms: (1) linear; (2) quadratic (INR + INR^2^), to test for non-linearity with a closed-form parabolic constraint; and (3) cubic B-splines (degree 3, df = 5, implemented via patsy bs() function in Python). Winsorization (1st–99th percentile) was applied before spline fitting to limit the influence of extreme values. All spline models were adjusted for age, organ dysfunctions, and lactate.

The spline models were preferred over the quadratic models because they imposed no parametric constraints and allowed the data to define the functional form. In contrast, quadratic models assumed symmetry, which may have misrepresented the INR–mortality relationship, particularly at higher INR values > 3.0.

Pointwise ORs (95% CI) along the spline curve were estimated using the delta method, defined as the contrast LP(x) − LP(reference). The LP represented the linear predictor with covariates fixed at their median values. The reference values were INR = 1.3 (observed mortality nadir) and platelet count = 200 × 10^9^/L.

Discriminative performance was assessed using AUROC. Model comparisons were based on incremental AUROC (ΔAUROC) over the base model (age + organ dysfunction). All analyses were stratified by the coagulation phenotype as described above.

### 2.5. Ethical Statement

The study was approved by the Ethics Committee of the Balearic Islands on 25 October 2023 (Approval No. 11/2023) and conducted in accordance with the Declaration of Helsinki. The requirement for written informed consent was waived due to the retrospective design and minimal risk to participants.

### 2.6. Declaration of Generative AI

The authors declare that Claude.ai model Opus 4.6 was used to assist with improving the clarity, grammar, and readability of this manuscript.

## 3. Results

### 3.1. Patient Characteristics

A total of 6433 patients with sepsis were included (60.6% females, 64.5 ± 16.7 years). The flowchart is shown in Supplemental Figure S1. Septic shock was diagnosed in 501 patients (7.8%). The overall mortality rate was 8.8%. ICU admission was required in 2028 patients (31.5%). Thrombocytopenia (<150 × 10^9^/L) was present in 1847 patients (28.7%). Non-survivors were significantly older than survivors (mean 71 vs. 63.8 years; *p* < 0.0001) and had higher INR values (median 1.4 vs. 1.3; *p* < 0.0001), lower platelet counts (182 vs. 199 × 10^9^/L; *p* = 0.0016), and higher rates of ICU admission (50.3% vs. 29.7%; *p* < 0.0001). Detailed clinical and laboratory characteristics are presented in Table 1.

Among the 1421 patients in the SIC cohort, in-hospital mortality was 12.1%. The distribution of coagulation phenotypes was as follows: mild/subthreshold alterations in 571 (40.2%, mortality 8.1%); an elevated INR only in 322 (22.7%, mortality 13.0%); thrombocytopenia only in 284 (20.0%, mortality 12.7%); and both altered (DIC pattern) in 244 (17.2%, mortality 19.7%). Additionally, 1914 patients were classified as having non-sepsis-related coagulopathy, with a pooled mortality of 9.9%. Among these, 998 patients (52.1%) had an INR ≥ 1.5 at activation, with an in-hospital mortality of 11.4%. Of these, 1273 (66.5%) patients had platelet counts < 150 × 10^9^/L (mortality 9.1%), and 357 patients (18.7%) met both criteria simultaneously (mortality 11.5%). The full cohort characteristics by coagulation phenotype are presented in Table 2.

### 3.2. Association Between the INR, Platelet Count, and In-Hospital Mortality

**Figure 1:**
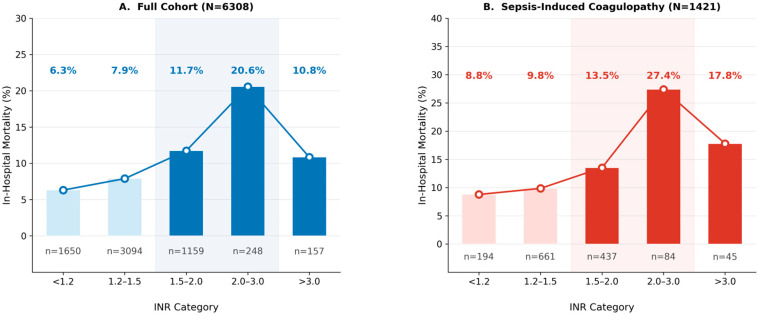
In-hospital mortality according to the INR category. (**A**) Full cohort (N = 6308). (**B**) Sepsis-Induced Coagulopathy (SIC) cohort (N = 1421). Bar height indicates crude in-hospital mortality (%). Line connects the category medians. The shaded region marks the pathological INR range (≥1.5). Percentage annotations above the bars indicate mortality and sample size per category.

The association between hemostatic parameters and in-hospital mortality was evaluated using clinical categories and adjusted models (Figure 1). As shown in Figure 1A, patients with an INR ≤ 1.2 had a mortality rate of 6.3%, which increased progressively to 20.2% in those with an INR of 2.0–3.0, followed by a moderate decline to 10.8% at INR > 3.0. This pattern indicated a non-linear relationship. In the SIC cohort, this gradient was more pronounced, with mortality increasing from 8.8% (INR < 1.2) to 27.4% (INR 2.0–3.0).

In contrast, the association between the platelet count and mortality was weaker and less well-defined. An increased risk was observed at the extreme values, particularly in patients with a platelet count < 50 × 10^9^/L (24.6% mortality), whereas the differences across the intermediate ranges were less pronounced compared to the INR. Local AUROC analysis showed that the platelet counts spline model performed poorly within its pathological range (<100 × 10^9^/L; local AUROC 0.57–0.68). In contrast, higher AUROC values in the normal platelet range (150–300 × 10^9^/L; local AUROC 0.75–0.80) likely reflected the influence of covariates rather than a direct platelet-driven prognostic signal. The platelet count showed limited prognostic value within its pathological range. The higher global AUROC in the full cohort was likely driven by variation in the normal range than a true pathological signal.

### 3.3. Non-Linear Modeling: Odds Ratio Along Spline Curve

**Figure 2:**
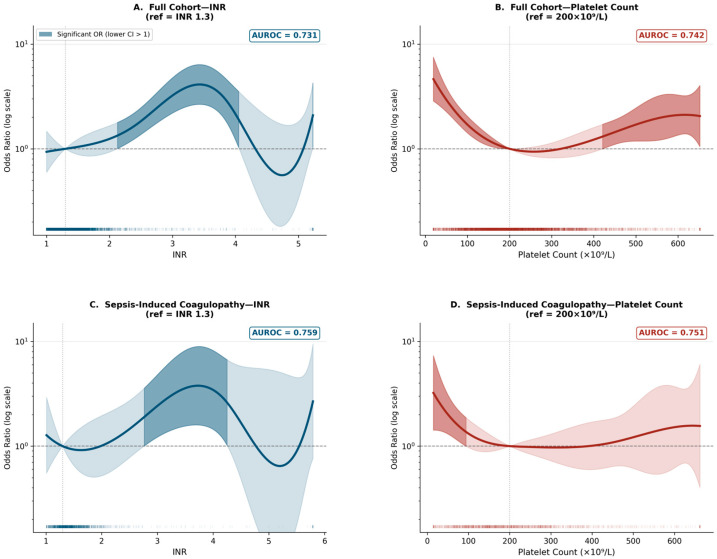
Odds ratio along the cubic B-spline curve for the INR and platelet count. (**A**) Full cohort for INR only; (**B**) full cohort for platelet count only; (**C**) Sepsis-Induced Coagulopathy for INR only; (**D**) Sepsis-Induced Coagulopathy for platelet count only. Reference: INR = 1.3; platelet count = 200 × 10^9^/L. Shaded region: 95% CI (delta method). Darker shading: region where lower CI > 1 (statistically significant elevation). Rug plot shows data density. Y-axis in log scale. AUROC shown in upper right corner of each panel.

To capture the full shape of the INR–mortality relationship without parametric constraints, cubic B-spline models (df = 5) were fitted and adjusted for age and non-coagulatory organ dysfunction. Figure 2 shows the estimated OR along the spline curve for the INR (panels A/C) and the platelet count (panels B/D) in the full cohort and the SIC cohort, respectively.

In the full cohort, the INR showed a significant non-linear association with mortality from an INR of 2.2 onwards (OR 1.41 [95% CI 1.06–1.87], *p* = 0.019), reaching a peak OR of 4.08 [2.64–6.32] at an INR of 3.5 (*p* < 0.001), followed by a decline above an INR of 4.0, consistent with the previously described U-shaped pattern. The platelet count was associated with significantly increased ORs below 175 × 10^9^/L, with the largest effect observed at <50 × 10^9^/L (OR 3.90 [2.66–5.74], *p* < 0.001).

In the SIC cohort, the INR–mortality association emerged at higher INR values: the OR became significant at an INR of 2.8 (OR 1.98 [1.04–3.76], *p* = 0.037) and reached an OR of 3.59 [1.56–8.24] at an INR of 3.5 (*p* = 0.003). For the platelet count in SIC, significant ORs were restricted to severe thrombocytopenia (<75 × 10^9^/L), with no significant association observed above 100 × 10^9^/L. The full OR tables at key clinical cut-off points are presented in Table 3.

### 3.4. Multivariable Models for In-Hospital Mortality Predictions

The interaction between the coagulation markers was evaluated by examining in-hospital mortality across the combined INR and platelet categories (Supplemental Figure S2). The INR was identified as the dominant predictor, with risks increasing progressively across the INR strata, irrespective of the platelet levels. Among patients with platelet counts within the normal range (150–400 × 10^9^/L), mortality increased from 5.1% (INR ≤ 1.2) to 21.1% (INR 2.0–3.0). In contrast, the platelet counts alone had a limited impact on mortality when the INR was elevated. In patients with an INR ≤ 1.2, thrombocytopenia provided additional risk stratification: mortality was 40% for platelet counts < 50 × 10^9^/L versus 5.1% for normal counts. The highest mortality (50%) occurred in patients with an INR > 2.0 and a platelet count < 50 × 10^9^/L.

### 3.5. Multivariable Models and Discriminative Performance

A multivariable logistic regression model was developed to identify independent predictors of in-hospital mortality. Table 4 presents model comparison across functional forms. The base model (age + Disf_sinCoag) achieved an AUROC of 0.716. In the full cohort, adding the INR as a linear term contributed marginally (ΔAUROC + 0.003, *p* = 0.017), whereas the quadratic model improved performance (ΔAUROC + 0.010). The cubic B-spline yielded the largest gain (ΔAUROC + 0.015), capturing non-linear effects not represented by the linear and quadratic terms. Adding the platelet count as a spline contributed ΔAUROC + 0.026 in the full cohort, but only ΔAUROC + 0.013 in the SIC cohort, less than the INR spline (ΔAUROC + 0.021 in SIC).

In the SIC cohort, the INR linear term was non-significant (*p* = 0.186), the quadratic term approached but did not reach significance (*p* = 0.067), and only the spline model captured the predictive signal (ΔAUROC + 0.021). These results indicated that the INR–mortality relationship in SIC was non-linear and required flexible modeling for detection.

In the extended multivariable analyses, including baseline coagulopathy (yes/no), hyperlactatemia (when available), and infection focus and bacteremia (dummy-coded when missing), the INR remained independently associated with in-hospital mortality. The discriminative performance of the model was unchanged (AUROC 0.726), and the effect size of the INR was stable (OR ≈ 1.09). Neither baseline coagulopathy (OR 0.994; *p* = 0.955) nor hyperlactatemia (OR 1.098; *p* = 0.444) attenuated this association. A sensitivity analysis excluding all patients with baseline coagulopathy preserved the characteristic inverted U-shaped relationship. Mortality for an INR > 3.0 remained markedly lower in patients without chronic coagulopathy (8.1%) compared with those with baseline conditions (18.2%). Adjusting for infection sources and bacteremia resulted in a slight improvement in discrimination (AUROC 0.736), with the INR retaining its independent prognostic effect (OR ≈ 1.08). These extended multivariable and sensitivity analyses confirmed that the prognostic value of the INR was robust and independent of early available confounders.

### 3.6. Discriminative Performance by the Coagulation Phenotype

**Figure 3:**
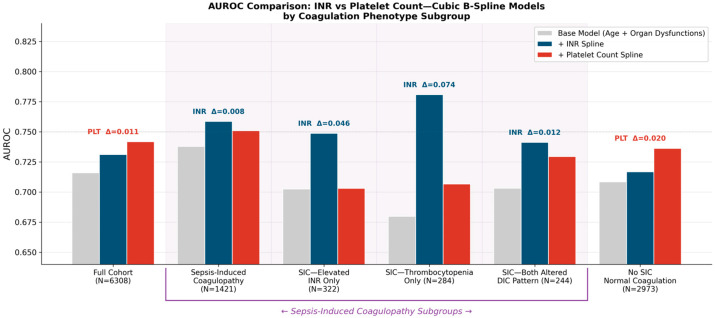
Discriminative performance (AUROC) by the coagulation phenotype. Grouped bar chart comparing AUROC of the base model (gray), INR spline (blue), and platelet count spline (red) across six subgroups: full cohort; SIC; SIC with elevated INR only; SIC with thrombocytopenia only; SIC with both altered (DIC pattern); and no SIC with normal coagulation. Labels above each group indicate the winning predictor and ΔAUROC between the INR and platelet splines.

Figure 3 shows AUROC values across the coagulation-phenotype subgroups. In the full cohort, the platelet-count spline demonstrated a slightly higher global AUROC than the INR (0.742 vs. 0.731). This pattern was reversed in the SIC cohort, where the INR spline outperformed the platelet count (0.759 vs. 0.751, ΔAUROC + 0.008). The superiority of the INR was consistent across all SIC phenotypic subgroups. In SIC patients with an isolated INR elevation (INR ≥ 1.5, platelet count ≥ 150 × 10^9^/L; N = 322), the INR spline achieved an AUROC of 0.748 compared with 0.703 for the platelet count (ΔAUROC + 0.045). In SIC patients with isolated thrombocytopenia (INR < 1.5, platelet count < 150 × 10^9^/L; N = 284), the INR spline showed an AUROC of 0.781 compared with 0.707 for the platelet count (ΔAUROC + 0.074). Within this subgroup, an INR ≥ 1.4, below the classical coagulopathy threshold, was associated with an OR of 7.3 (95% CI 2.3–23.4, *p* = 0.001). This indicated that even a subclinical INR elevation carried significant prognostic value in thrombocytopenic patients. In the DIC-pattern subgroup (both altered; N = 244), the INR also demonstrated moderate superiority (AUROC 0.742 vs. 0.729).

To contextualize the performance of the INR spline model against an established composite score, it was compared with the SIC score (range 0–6), which incorporated the platelet count, a dichotomized INR (thresholds 1.2 and 1.4), and the SOFA. In the full cohort, the continuous SIC score achieved an AUROC of 0.733 (Brier Skill Score 0.061), which was marginally higher than the INR spline alone (AUROC 0.731, Skill = 0.062). When the dichotomized INR component of the SIC score was replaced with the continuous INR spline, while retaining the platelet and SOFA components, the discrimination improved to an AUROC of 0.738. The continuous NRI increased by +0.039 over the original SIC score (driven by +0.104 in non-events). In the SIC cohort, the same substitution resulted in an AUROC of 0.760 compared with 0.754 for the original SIC score, yielding an NRI of +0.097 (events + 0.046, non-events + 0.050). The likelihood ratio test confirmed that the INR spline did not contribute significant additional information beyond the complete SIC score (*p* = 0.065 for the full cohort and *p* = 0.572 for the SIC cohort). This finding was consistent with the fact that the INR was already incorporated into the score, albeit sub-optimally through dichotomization. Calibration plots (Supplemental Figure S3B,E) showed that the INR spline model tracked more closely with the observed mortality than the SIC score across most deciles, particularly in the intermediate risk range.

### 3.7. Subgroup Analyses

To assess the consistency of the INR as a prognostic marker, stratified logistic regression analyses were performed across age, sex, ICU admission, septic shock, care setting, number of organ dysfunctions, and coagulopathy status (Supplemental Table S1 and Supplemental Figure S4). The INR remained significantly associated with in-hospital mortality in the overall cohort (OR 1.13; 95% CI 1.04–1.23), with stronger associations observed in patients younger than 65 years (OR 1.81; 95% CI 1.40–2.36), in females (OR 1.23; 95% CI 1.02–1.46), and in non-ICU patients (OR 1.16; 95% CI 1.04–1.27). A significant association was also observed in patients with acute coagulopathy attributed to sepsis (OR 1.18; 95% CI 1.02–1.36), whereas the association was less pronounced in individuals with pre-existing coagulation conditions.

## 4. Discussion

This study showed that the INR measured at the time of sepsis diagnosis was an independent predictor of in-hospital mortality and exhibited a non-linear association with risk. Within the clinically relevant context of SIC, the INR showed greater discriminative value than the platelet count. These findings extended previous work by applying flexible non-linear modeling to characterize the full shape of the INR–mortality relationship. Within this cohort, the INR outperformed platelet counts across all coagulation phenotypic subgroups of SIC, including subgroups in which thrombocytopenia, rather than an elevated INR, constituted the predominant abnormality.

These findings should be interpreted in light of the methodological constraints. Although multiple sensitivity analyses were performed, residual confounding cannot be excluded given the retrospective, single-center design. Furthermore, although the association between the INR and mortality was statistically robust and consistent across subgroups, its discriminative performance remained moderate. Consequently, the INR should be regarded as an early risk enrichment marker that can complement but not replace existing prognostic tools.

Previous studies have explored coagulopathy in sepsis using composite scores such as ISTH overt-DIC or the SIC, both of which incorporated the INR among other parameters [6,22,23]. However, the role of the INR as an independent prognostic marker has only been examined to a limited extent. A recent study [24] reported that an INR > 1.5 was associated with an increased 30-day mortality. Zhang et al. [25] showed an INR > 1.22 predicted sepsis severity even in the absence of an underlying disease. Jo et al. [26] observed that failure to correct coagulopathy (defined as INR > 1.5 or platelet count < 50 × 10^9^/L) was associated with a delayed source control and a significantly higher 28-day mortality. However, most of these studies were conducted in ICU populations and did not compare the INR and platelet count using the same analytical framework within a single cohort. The differences in cohort assembly could also complicate interpretation. The present study used a hospital-wide protocol based on a modified Sepsis-2 activation system, which ensured early identification and standardized sampling at diagnosis. This design enhanced internal consistency, but it may have limited direct comparability with Sepsis-3-defined cohorts and could contribute to observational differences in the effect sizes across studies.

The superiority of the INR spline over the platelet count in SIC has mechanistical plausibility. The INR reflected activation of the extrinsic coagulation pathway and hepatic synthetic function, both of which can be directly affected by sepsis-induced cytokine release and microvascular dysfunction [13,14]. Its elevation therefore can provide an early and direct signal of hemostatic disturbance. In contrast, the platelet count may decrease through several mechanisms, including consumption, sequestration, hemodilution, or pre-existing thrombocytopenia. In the absence of baseline conditions (as in SIC), thrombocytopenia must reach values below 75 × 10^9^/L to provide independent prognostic information. Such levels were more typical of established rather than early coagulopathy. By comparison, the INR reached its prognostic threshold (1.5) earlier during sepsis, consistent with clinical observations. These mechanistic explanations cannot exclude potential bias. The timing and indications for laboratory testing, as well as unmeasured comorbidities, may have still influenced the observed associations.

The observation that the INR predicted mortality even in SIC patients whose dominant abnormality was thrombocytopenia (AUROC 0.781 vs. 0.707, ΔAUROC + 0.074) was particularly noteworthy. In this subgroup, an INR ≥ 1.4 (a value below the conventional coagulopathy threshold) was associated with a seven-fold increase in mortality (OR 7.3, *p* = 0.001). This finding suggested that the INR captured a component of hemostatic dysfunction not reflected by the platelet count. It also indicated that a subclinical INR elevation may serve as an early warning signal, even when thrombocytopenia is the primary abnormality. Given the smaller sample size in these subgroups and the wider CIs, these effects estimates should be interpreted with caution and validated prospectively.

The non-linear relationship between the INR and mortality (characterized by a progressive increase from an INR of 1.5 to 3.5 followed by a decline) was consistent across all analytical approaches used in this study. The attenuation of risk at INR values above 4.0 reflected patient heterogeneity. At these very high INR levels, a proportion of patients may have pre-existing anticoagulation, chronic liver disease, or vitamin K deficiency rather than sepsis-driven coagulopathy. These conditions may not be fully recognized at the time of sepsis diagnosis. This interpretation was supported by a steeper and more sustained INR–mortality gradient observed in the SIC cohort, where such baseline conditions have been excluded by definition. The cubic B-spline framework was well-suited for capturing this non-monotonic pattern, as it did not impose a predefined shape; instead, the pattern emerged directly from the data. Incomplete identification of prior anticoagulant exposure or underlying liver dysfunction at activation could still contribute to the attenuated risk at very high INR values. These considerations have highlighted the need for careful phenotyping in future studies.

The findings reinforced the INR as an early marker of coagulation dysfunction in sepsis, reflecting both hepatic impairment and cytokine-driven activation of the extrinsic pathway [7,8,9]. Unlike the platelet count, which may decrease through multiple mechanisms, an INR elevation can occur earlier and provide a more direct indication of hemostatic disturbance. This has practical relevance, as SOFA includes the platelet count but omits the INR, limiting the early detection of coagulation abnormalities. The transition from Sepsis-2 to Sepsis-3 may contribute to this limitation, as the INR is no longer included as a marker of organ dysfunction—this could reduce sensitivity to early coagulation abnormalities. The exclusion of the INR from Sepsis-3 definitions does not indicate a limitation of the framework, although it may reduce sensitivity to certain early coagulation disturbances. 

Given the retrospective design and the moderate discriminative performance, these findings should be considered hypothesis-generating rather than prescriptive. The results support reconsideration of the INR in future revisions of sepsis definitions and scoring systems. Such reconsideration would require prospective validation, external replication across diverse care settings, and formal evaluation of clinical utility and calibration before guideline integration.

The practical applicability of SOFAs in hospital-wide settings has been limited by its requirement for continuous numerical variables, most notably with the Glasgow Coma Scale. This variable is not systematically available outside the ICU at the time of sepsis recognition. Sepsis-2-based systems, including the protocol used in this study, can capture level-of-consciousness impairment qualitatively, enabling early identification across all care settings. This aligned with real-world practice, where according to the European Sepsis Care Survey, fewer than half of European hospitals have applied Sepsis-3 definitions clinically. This supported the validity and relevance of Sepsis-2-based hospital-wide implementation strategies. Within this pragmatic context, the INR may contribute to early risk stratification; however, it should be used alongside, rather than in place of, established clinical assessment and organ dysfunction scoring.

Early disturbances in coagulation may have influenced clinical decision-making beyond emergency and critical care settings, including cardiology and internal medicine. Infection-related coagulopathy has been shown to delay cardiac surgery until infection has been controlled. This illustrated how early recognition of sepsis-associated coagulation abnormalities can directly affect therapeutic planning [27]. These considerations have highlighted that simple and widely available parameters such as the INR may provide clinically actionable information across multiple medical specialties, extending the relevance of these findings beyond traditional sepsis care pathways.

Unlike composite scores that require specialized tests or less accessible biomarkers, the INR is a standardized, low-cost, and universally available biomarker. It is part of routine blood panels and can be interpreted immediately at all care levels, making it ideal for early risk stratification, particularly in emergency settings where rapid treatment decisions and ICU prioritization are critical. The results supported the pragmatic use of the INR to identify high-risk patients early, while underscoring the need for prospective studies to determine its incremental clinical value, as well as its impact on decision-making and safety when integrated into triage pathways.

### Limitations

This study has some limitations. Its retrospective, single-center design may limit the generalizability. Although some baseline variables showed statistically significant differences between survivors and non-survivors, many of these contrasts were small in magnitude and should be interpreted with clinical caution. These differences did not drive the prognostic conclusions, which relied instead on the non-linear multivariable models and on context-dependent analyses such as SIC classification via INR × platelet profiles. The use of a modified Sepsis-2 protocol, while effective for early detection, may not have fully aligned with Sepsis-3 criteria; however, this protocol has previously demonstrated strong performance in early sepsis identification [18].

A sensitivity analysis excluding patients with pre-existing causes of coagulopathy (chronic liver disease or anticoagulant therapy) confirmed that the association between the INR and mortality was not driven by baseline coagulation disorders. This supported the validity of the INR as a marker of sepsis-induced hemostatic dysfunction. Additionally, composite scores such as the ISTH overt-DIC could not be fully calculated because D-dimer measurements were not available at sepsis diagnosis. This marker has not been routinely obtained during early sepsis assessment, which has highlighted the practical utility of the INR as a widely accessible early parameter. The SOFA score could not be calculated directly because it required continuous numerical variables, such as the Glasgow Coma Scale, which were not systematically available in the dataset. However, the protocol used in this study captured level-of-consciousness impairment qualitatively, consistent with criteria applied in Sepsis-2-based systems, and was incorporated into structured sensitivity analyses. Furthermore, the 2024 European Sepsis Care Survey [28] reported that fewer than half of European hospitals have applied Sepsis-3 definitions in routine clinical practice. This reinforced the ongoing relevance of Sepsis-2-based protocols for real-world hospital-wide implementation.

The definition of SIC in this study was clinician-assigned rather than based on a formal scoring system such as the ISTH or SIC score. Although this approach may have reduced standardization, it reflected real-world clinical reasoning and added ecological validity. Prior anticoagulant use had not been adjusted for, as the objective was to evaluate the prognostic value of the INR at the time of diagnosis, irrespective of its underlying cause. All AUROC values represented in-sample estimates, and external validation in an independent cohort will be required to confirm the generalizability of the spline models. The SIC phenotype subgroup analyses should be considered exploratory, given the smaller sample sizes (N = 244–322) and the associated wide CI. These findings should warrant cautious interpretation and prospective replication.

Despite these limitations, this study benefited from one of the largest hospital-wide sepsis cohorts reported to date, a structured protocol active since 2006 that enabled pre-failure biomarker assessments, and from methodologically rigorous flexible modeling that avoided the parametric constraints used in earlier analyses.

## 5. Conclusions

The INR measured at sepsis diagnosis was independently and non-linearly associated with in-hospital mortality, demonstrating greater prognostic value than the platelet count. This superiority was most evident within the SIC subgroup, where the INR spline models outperformed the platelet count across all coagulation phenotypes, including isolated thrombocytopenia. The non-linear relationship required flexible modeling to be captured accurately, while linear models underestimated the prognostic signal, particularly in SIC.

The predictive strength of the INR across clinical settings indicated its potential as an early risk stratification marker. Incorporating the INR into triage protocols and future sepsis guidelines could improve the early recognition of high-risk patients and enhance clinical decision-making.

## Figures and Tables

**Table 1:** Baseline and clinical characteristics in septic patients by hospital mortality.

KERRYPNX	Total (N = 6433)	Survivors (N = 5866)	Non-Survivors (N = 567)	*p*-Value
**Age, mean (SD)**	64.5 ± 16.7	63.8 ± 16.9	71 ± 13.2	**<0.0001**
**Sex, n (%)**				0.1373
Male	2529 (39.3)	2323 (39.6)	206 (36.3)	
Female	3904 (60.6)	3543 (60.4)	361 (63.7)	
**Laboratory values, median [IQR]**				
Platelets, ×10^9^/L	197 [141–277]	199 [144–277]	182 [113–283]	**0.0016**
Bilirubin, mg/dL	0.6 [0.4–1.1]	0.6 [0.4–1.1]	0.8 [0.4–1.5]	**<0.0001**
WBC count, ×10^9^/L	11.9 [7.7–17.1]	11.9 [7.7–17.1]	11.9 [6.9–18.2]	0.8847
Fibrinogen, mg/dL	736 [598–867.5]	740 [603–871]	698 [524.5–832.5]	**<0.0001**
C-reactive protein, mg/L	170 [86.9–245.7]	169.6 [85.8–245.3]	174.7 [94.1–257.6]	0.1060
PCT, ng/mL	2.1 [0.4–9.7]	2 [0.4–9.4]	3.3 [0.7–13.6]	**<0.0001**
Cholesterol, mg/dL	120 [96–147]	121 [97–147]	105 [82–136]	**<0.0001**
INR	1.3 [1.1–1.4]	1.3 [1.1–1.4]	1.4 [1.2–1.6]	**<0.0001**
**ED admission, n (%)**	2522 (39.2)	2369 (40.3)	153 (26.9)	**<0.0001**
**ICU admission, n (%)**	2028 (31.5)	1743 (29.7)	285 (50.3)	**<0.0001**
**ICU stay (days), median [IQR]**	9 [6–17]	9 [6–16]	20 [8–39]	**<0.0001**
**Overall length of stay (days), median [IQR]**	9 [6–17]	9 [6–16]	14 [5–27]	**<0.0001**
**Thrombocytopenia, n (%)**				
**yes**	1847 (28.7)	1640 (27.9)	207 (36.5)	**<0.0001**
**no**	4586 (71.3)	4226 (72.1)	360 (63.5)	

**Bold** values indicate statistically significant differences (*p* ≤ 0.05). Abbreviations: ED: Emergency Department; ICU: Intensive Care Unit; INR: International Normalized Ratio; IQR: Interquartile Range; PCT: Procalcitonin; SD: Standard Deviation; WBC: White Blood Cell.

**Table 2:** Cohort characteristics by coagulation phenotype.

Coagulation Phenotype	Full Cohort	Mild/Subthreshold	Elevated INR	Thrombocytopenia	Both Altered (DIC Pattern)	SIC (COAGULO = 1)
N	6308	3544	963	1200	601	1421
Mortality, %	**8.8**	**6.7**	**11.9**	**9.2**	**14.8**	**12.1**
Age, median [IQR]	68 [54–77]	67 [53–76]	70 [56–79]	68 [55–78]	68 [56–77]	65 [50–76]
INR, median [IQR]	1.31 [1.1–1.4]	1.25 [1.1–1.3]	1.71 [1.5–2.01]	1.26 [1.1–1.3]	1.71 [1.6–2.02]	1.43 [1.2–1.6]
Platelets (×10^9^/L), median [IQR]	198 [142–278]	240 [191–314]	233 [183–312]	111 [83–134]	103 [66–132]	185 [112–279]
Organ dysfunctions, median [IQR]	1 [1–2]	1 [1–2]	1 [1–2]	1 [1–2]	2 [1–3]	1 [0–2]

Mild/subthreshold is defined as INR of 1.2–1.5. Elevated INR is defined as INR ≥ 1.5. Thrombocytopenia is defined as platelet count < 150 × 10^9^/L. Both altered is defined as INR ≥ 1.5 and platelet count < 150 × 10^9^/L. Organ dysfunctions are defined as sum of seven non-coagulatory criteria (hypotension, hypoxaemia, oliguria, altered consciousness, elevated creatinine, hyperbilirubinaemia, hyperlactataemia). SIC (COAGULO = 1) is defined as episodes where coagulation abnormality was attributed to sepsis by the treatment clinician. Bold font and red text indicate values of particular clinical relevance. Abbreviations: DIC: Disseminated Intravascular Coagulation; INR: International Normalized Ratio; IQR: Interquartile Range; SIC: Sepsis-Induced Coagulopathy.

**Table 3:** Odds ratios along the cubic B-spline curve at key clinical cut-points.

Value	Odds Ratio	95% Confidence Interval	*p*-Value	
**Full Cohort—INR (ref = INR 1.3, AUROC = 0.731)**				
1.3	**1.001**	[0.997–1.004]	**0.8593**	
1.5	**1.045**	[0.882–1.238]	**0.6129**	
1.8	**1.140**	[0.865–1.503]	**0.3504**	
2	**1.245**	[0.932–1.662]	**0.1373**	
2.5	**1.841**	[1.372–2.470]	**<0.001**	***
3	**3.232**	[2.195–4.759]	**<0.001**	***
3.5	**4.085**	[2.642–6.316]	**<0.001**	***
4	**2.146**	[1.219–3.780]	**0.0082**	**
**Full Cohort—Platelet Count (×10^9^/L) (ref = 200 × 10^9^/L, AUROC = 0.742)**				
200	**0.999**	[0.999–1.000]	**0.8008**	
150	**1.202**	[1.071–1.350]	**0.0019**	**
100	**1.699**	[1.418–2.035]	**<0.001**	***
75	**2.172**	[1.780–2.650]	**<0.001**	***
50	**2.934**	[2.255–3.818]	**<0.001**	***
30	**3.897**	[2.650–5.730]	**<0.001**	***
**Sepsis-Induced Coagulopathy—INR (ref = INR 1.3, AUROC = 0.759)**				
1.3	**0.999**	[0.993–1.004]	**0.8702**	
1.5	**0.927**	[0.652–1.320]	**0.6745**	
1.8	**0.936**	[0.508–1.724]	**0.8324**	
2	**1.009**	[0.521–1.955]	**0.9782**	
2.5	**1.458**	[0.779–2.730]	**0.2380**	
3	**2.424**	[1.221–4.810]	**0.0113**	*
3.5	**3.578**	[1.558–8.218]	**0.0027**	**
4	**3.438**	[1.445–8.180]	**0.0052**	**
**Sepsis-Induced Coagulopathy—Platelet Count (×10^9^/L) (ref = 200 × 10^9^/L, AUROC = 0.751)**				
200	**1.000**	[0.998–1.002]	**0.8770**	
150	**1.073**	[0.889–1.294]	**0.4641**	
100	**1.316**	[0.972–1.782]	**0.0761**	
75	**1.573**	[1.123–2.204]	**0.0084**	**
50	**2.007**	[1.306–3.084]	**0.0015**	**
30	**2.574**	[1.403–4.721]	**0.0023**	**

Cubic B-spline models (df = 5) adjusted for age and non-coagulator organ dysfunction count. OR estimated at each value versus the reference using the delta method. Bold font and color text indicate values of particular clinical relevance. Reference: INR of 1.3 (observed mortality nadir); platelet count of 200 × 10^9^/L. Green shading: *p* < 0.05. Gray shading: reference value. * *p* < 0.05 ** *p* < 0.01 *** *p* < 0.001.

**Table 4:** Model comparisons—functional form and discriminative performance.

Model	AUROC	ΔAUROC	INR— OR or Form	*p* (INR)	Platelets—OR or Form	*p* (Platelets)
**Full Cohort (N = 6308, mortality = 8.8%)**						
Age + Organ dysfunctions	**0.716**	—	—	—	—	—
Base + INR (linear)	**0.719**	**+0.003**	1.116	**0.0166**	—	—
Base + INR + INR^2^ (quadratic)	**0.726**	**+0.010**	1.747 at INR 2.5	**<0.001**	—	—
***Base + INR (cubic B-spline, df = 5)***	**0.731**	**+0.015**	see Figure 2	non-linear	—	—
*Base + Platelet count (cubic B-spline, df = 5)*	**0.742**	**+0.026**	—	—	see Figure 2	non-linear
***Base + INR spline + Platelet spline (joint)***	**0.751**	**+0.035**	see Figure 2	non-linear	see Figure 2	non-linear
**Sepsis-Induced Coagulopathy (N = 1421, mortality = 12.1%)**						
Age + Organ dysfunctions	**0.738**	—	—	—	—	—
Base + INR (linear)	**0.743**	**+0.006**	1.111	0.1855	—	—
Base + INR + INR^2^ (quadratic)	**0.75**	**+0.013**	1.747 at INR 2.5	0.0667	—	—
***Base + INR (cubic B-spline, df = 5)***	**0.759**	**+0.021**	see Figure 2	non-linear	—	—
*Base + Platelet count (cubic B-spline, df = 5)*	**0.751**	**+0.013**	—	—	see Figure 2	non-linear
***Base + INR spline + Platelet spline (joint)***	**0.767**	**+0.029**	see Figure 2	non-linear	see Figure 2	non-linear

All models adjusted for age and non-coagulator organ dysfunction count (base model). ΔAUROC = increment over base model. INR quadratic model: OR at INR of 2.5 vs. reference of 1.3, derived from linear + quadratic coefficients. Spline models (df = 5): OR estimated pointwise; see Figure 2 for full curves. Bold font and color text indicate values of particular clinical relevance. The progression from linear to quadratic to spline shows that the INR–mortality relationship is non-linear—the linear model underestimates the predictive contribution of INR, while the spline captures the full signal. Blue shading = INR-based models; red shading = platelet-based; green = joint model.

## Data Availability

The data that support the findings of this study are not openly available due to reasons of sensitivity and are available from the corresponding authors (AG) upon request. Data are stored under controlled access at the Sepsis Unit of Son Llàtzer University Hospital (Spain).

## Supplementary Materials

The following supporting information can be downloaded at: https://www.mdpi.com/article/10.3390/biomedicines14040839/s1, Section S1: Sepsis Protocol Activation System; Table S1: Prognostic value of INR in subgroup analyses. CI: Confidence Interval; ED: Emergency Department; ICU: Intensive Care Unit; INR: International Normalized Ratio; OR: Odds Ratio. Bold values indicate statistically significant differences (*p* ≤ 0.05); Figure S1: Flowchart of patient selection and study population; Figure S2: Joint in-hospital mortality (%) by INR × platelet count category. Full cohort (N = 6308). Columns: INR categories (<1.2, 1.2–1.5, 1.5–2.0, 2.0–3.0, >3.0). Rows: Platelet count categories (<50, 50–100, 100–150, 150–200, 200–300, >300 × 10^9^/L). Color scale: 0–35% mortality. Cells with N < 10 shown in gray with italicized annotation. Color intensity reflects mortality rate (darker = higher risk). Cells with N < 10 are excluded. This figure provides contextual visualization of the joint risk surface and supports the interpretation that the INR strata dominate the mortality gradient regardless of the platelet count level; Figure S3: Comparison of the INR cubic B-spline model with the Sepsis-Induced Coagulopathy (SIC) score for predicting in-hospital mortality. Upper row: Full cohort (N = 6308, mortality 8.8%). Lower row: Sepsis-Induced Coagulopathy subgroup (N = 1421, mortality 12.1%). Panel A/D: ROC curves for the base model (age + organ dysfunctions, gray dashed), SIC score continuous 0–6 (orange), INR cubic B-spline df = 5 (blue), and a modified model replacing the SIC score’s dichotomized the INR component with the continuous INR spline while retaining the platelet and SOFA components (SIC coag + SOFA + INR spline, green). Panel B/E: calibration plots by predicted probability decile; each point represents the mean predicted probability (x-axis) versus observed mortality (y-axis) within each decile; the dashed diagonal represents perfect calibration. Panel C/F: AUROC comparison across the four models; values above bars indicate AUROC and increment over the base model (Δ); inset shows continuous NRI, IDI, and likelihood ratio test *p*-value for the modified versus original SIC score. All logistic regression models adjusted for age and non-coagulatory organ dysfunction count (Disf_sinCoag). INR winsorized at the 1st–99th percentile prior to spline fitting; Figure S4: Subgroup analysis of INR as a prognostic marker for in-hospital mortality. Forest plot displaying odds ratios (OR) and 95% confidence intervals for the association between INR and in-hospital mortality across clinical subgroups including age (14–29, 30–44, 45–59, 60–74, and ≥75 years old), sex (male/female), ICU admission (yes/no), septic shock (yes/no), care settings (emergency department, hospital ward, ICU), clinical severity as number of dysfunctions (low as 0–1 dysfunctions, severe as 2+ dysfunctions), and coagulopathy status (yes/no). Models were adjusted for relevant covariates within each subgroup.

## Abbreviations

The following abbreviations are used in this manuscript:

CI	Confidence Interval
CRP	C-Reactive Protein
DIC	Disseminated Intravascular Coagulation
ICU	Intensive Care Unit
INR	International Normalized Ratio
IQR	Interquartile Range
ISTH	International Society on Thrombosis and Hemostasis
OR	Odds Ratio
PCT	Procalcitonin
SIC	Sepsis-Induced Coagulopathy
SD	Standard Deviation
SOFA	Sequential Organ Failure Assessment
