# Supplementary material for: Prognostic Value of International Normalized Ratio and Thrombocytopenia in Early Risk Stratification of Septic Patients
Source: Biomedicines. 2026 Apr 7;14(4):839. doi: 10.3390/biomedicines14040839 (PMC13112952; doi:10.3390/biomedicines14040839)
Supplement: Supplementary file 1 [file biomedicines-14-00839-s001.zip › biomedicines-4118132-supplementary.pdf]

Supplementary information for:

Prognostic Value of International Normalized Ratio and Thrombocytopenia in Early Risk Stratification of Septic Patients

Authors

Sofía Tejada[1], Andrés Giglio[1,2,3,4*], Maria Aranda[1,5], Antonia Socias[1,5,6], Alberto del Castillo[1,5], Joana Mena[1], Sara Franco[1,5], Maria Ortega[1,5], Yasmina Nieto[1,5], Marcio Borges-Sa [1,2,5,6]

Affiliations

[1] Multidisciplinary Sepsis Group, Health Research Institute of the Balearic Islands (IdISBa), 07120 Palma de Mallorca, Spain

[2] Fundación Código Sepsis, 46003 Valencia, Spain

[3] Critical Care Department, Clinica Las Condes Hospital, 7591046 Santiago, Chile

[4] Critical Care Department, Finis Terrae University, 7640471 Santiago, Chile

[5] Multidisciplinary Sepsis Unit, Intensive Care Unit, Son Llàtzer Hospital, 07198 Palma de Mallorca, Spain

[6] Faculty of Medicine, Balearic Islands University (UIB), 07122 Palma de Mallorca, Spain

***Corresponding authors:** Andrés Giglio (agiglioj@gmail.com)

Table of contents:

Supplemental Section S1. Sepsis Protocol Activation System

Supplemental Table S1. Prognostic value of INR in subgroup analyses

Supplemental Figure S1. Flowchart of Patient Selection and Study Population

Supplemental Figure S2. Supplementary Figure S1. Joint in-hospital mortality (%) by INR × platelet count category. Full cohort (N=6,308). Columns: INR categories (<1.2, 1.2–1.5, 1.5–2.0, 2.0–3.0, >3.0). Rows: platelet count categories (<50, 50–100, 100–150, 150–200, 200–300, >300 $\times 10^9$/L). Colour scale: 0–35% mortality. Cells with N<10 shown in grey with italicised annotation. Colour intensity reflects mortality rate (darker = higher risk). Cells with N<10 are excluded. This figure provides contextual visualisation of the joint risk surface and supports the interpretation that INR strata dominate the mortality gradient regardless of platelet count level.

Supplementary Figure S3. Comparison of the INR cubic B-spline model with the SIC score for predicting in-hospital mortality. Upper row: full cohort (N=6,308, mortality 8.8%). Lower row: SIC subgroup (N=1,421, mortality 12.1%). **Panel A/D:** ROC curves for the base model (age + organ dysfunctions, grey dashed), SIC score continuous 0–6 (orange), INR cubic B-spline df=5 (blue), and a modified model replacing the SIC score's dichotomised INR component with the continuous INR spline while retaining the platelet and SOFA components (SIC coag+SOFA + INR spline, green). **Panel B/E:** calibration plots by predicted probability decile; each point represents the mean predicted probability (x-axis) versus observed mortality (y-axis) within each decile; the dashed diagonal represents perfect calibration. **Panel C/F:** AUROC comparison across the four models; values above bars indicate AUROC and increment over the base model (Δ); inset shows continuous NRI, IDI, and likelihood-ratio test p-value for the modified vs original SIC score. All logistic regression models adjusted for age and non-coagulatory organ dysfunction count (Disf_sinCoag). INR winsorised at the 1st–99th percentile prior to spline fitting.

Supplemental Figure S4. Subgroup analysis of INR as prognostic marker for in-hospital mortality. Forest plot displaying odds ratios (OR) and 95% confidence intervals for the association between INR and in-hospital mortality across clinical subgroups including age (14-29, 30-44, 45-59, 60-74, and ≥75 years old), sex (male/female), ICU admission (yes/no), septic shock (yes/no), care settings (emergency department, hospital ward, ICU), clinical severity as number of dysfunctions (low as 0-1 dysfunctions, severe as 2+ dysfunctions), and coagulopathy status (yes/no). Models were adjusted for relevant covariates within each subgroup.

Supplemental Section S1. Sepsis Protocol Activation System

Sepsis identification in this study was conducted through a structured, hospital-wide protocol implemented since 2006, designed to facilitate early recognition and standardized management of patients with suspected sepsis or septic shock. The code can be voluntarily activated either by the attending clinicians or by the Multidisciplinary Sepsis Unit (MSU). This expert unit comprises a multidisciplinary team of physicians and nurses, including specialists in critical care, internal medicine, infectious diseases, microbiologists, pharmacy, and sepsis management, along with clinical personnel from all relevant departments, such as the emergency department (ED).

Once the sepsis code is activated upon suspicious of sepsis or septic shock, all cases are prospectively evaluated by the MSU. The operational details of this protocol have been previously described. While Sepsis-3 definitions are used for final classification, the operational protocol relies on a modified version of Sepsis-2, requiring the presence at least one organ dysfunction and at least one systemic inflammatory response syndrome (SIRS) criterion. This approach has demonstrated strong performance in early sepsis detection through internal validation and extensive clinical experience. While Sepsis-3 emphasizes overt organ failure, it may delay recognition in patients with early hemostatic disturbances, such as coagulopathy. Therefore, the modified Sepsis-2 protocol remains a valuable tool for timely risk stratification, particularly in ED settings where rapid decision-making is critical.

Although this study used a severe sepsis–based protocol derived from Sepsis 2, this system includes organ dysfunction criteria equivalent to the components of SOFA, incorporating respiratory, hemodynamic, renal, neurological, and coagulation abnormalities (including INR). This structure enables early identification of organ dysfunction aligned with Sepsis 3 principles, within the operational framework of the hospital wide protocol implemented at our center since 2006.

The rationale for this dual approach lies in the need to identify early pathophysiological disturbances, such as hemostatic dysfunction, before overt organ failure occurs. By allowing protocol activation at the point of clinical suspicion, this system enables earlier prognostic assessment and timely therapeutic intervention. This is particularly relevant in ED settings, where rapid decision-making is essential and traditional criteria may delay recognition of high-risk patients.

Supplemental Table S1. Prognostic value of INR in subgroup analyses

Outcome	Subgroup	N	Events	%	INR model	OR	95% CI	p-value
Mortality	Age 14-29	268	3	1.1	ref	-	-	-
Mortality	Age 30-44	604	19	3.1	Lineal	7.12	2.26-22.43	**<0.001**
Mortality	Age 45-59	1255	85	6.8	Lineal	2.16	1.47-3.16	**<0.001**
Mortality	Age 60-74	2244	190	8.5	Quadratic	4.02	1.73-9.31	**0.0124**
Mortality	Age +75	2062	270	13.1	Quadratic	1.89	1.22-2.93	**0.0112**
Mortality	Male	3904	361	9.2	Quadratic	2.35	1.6-3.46	**<0.001**
Mortality	Female	2529	206	8.1	Quadratic	9.16	3.78-22.22	**<0.001**
Mortality	ED	2522	153	6.1	Quadratic	2.52	1.47-4.34	**0.0155**
Mortality	Ward	1883	129	6.9	Quadratic	3.26	1.73-6.14	**0.0073**
Mortality	ICU	2028	285	14.1	Quadratic	8.56	3.51-20.88	**<0.001**
Mortality	0–1 Dysfunctions	5448	401	7.4	Quadratic	2.2	1.52-3.19	**0.0021**
Mortality	≥2 Dysfunctions	985	166	16.9	Quadratic	6.24	2.49-15.65	**0.0029**
ICU admission	Age 14-29	268	62	23.1	Quadratic	0	0-14.25	0.0936
ICU admission	Age 30-44	604	141	23.3	Lineal	2.19	1.13-4.26	**0.0203**
ICU admission	Age 45-59	1255	350	27.9	Quadratic	5.49	2.03-14.89	**0.0149**
ICU admission	Age 60-74	2244	674	30	Quadratic	2.14	1.23-3.75	**0.0214**
ICU admission	Age +75	2062	438	21.2	Quadratic	1.48	1.02-2.17	**0.0310**
ICU admission	Male	3904	1053	27	Quadratic	1.42	1.04-1.96	**0.0139**
ICU admission	Female	2529	612	24.2	Quadratic	3.83	2.21-6.63	**<0.001**
ICU admission	ED	2522	377	14.9	Quadratic	1.56	1.05-2.32	0.0837
ICU admission	Ward	1883	272	14.4	Quadratic	6.52	2.57-16.5	**0.0013**
ICU admission	ICU	2028	1016	50.1	Quadratic	3.14	1.82-5.42	**0.0011**
ICU admission	0–1 Dysfunctions	5448	1252	23	Quadratic	1.33	1.01-1.76	**0.0302**
ICU admission	≥2 Dysfunctions	985	413	41.9	Quadratic	9.48	4.13-21.75	**<0.001**
Septic shock	Age 14-29	268	7	2.6	Lineal	1.02	0.05-21.26	0.9874
Septic shock	Age 30-44	604	25	4.1	Lineal	3.88	1.26-11.9	**0.0178**
Septic shock	Age 45-59	1255	87	6.9	Lineal	1.26	0.82-1.96	0.2923
Septic shock	Age 60-74	2244	169	7.5	Quadratic	6.96	2.7-17.89	**0.0011**
Septic shock	Age +75	2062	213	10.3	Quadratic	1.68	1.08-2.61	0.0507
Septic shock	Male	3904	304	7.8	Quadratic	1.89	1.26-2.82	0.0186
Septic shock	Female	2529	197	7.8	Quadratic	10.27	3.87-27.23	**<0.001**
Septic shock	ED	2522	164	6.5	Lineal	1.13	0.97-1.32	0.1177
Septic shock	Ward	1883	102	5.4	Quadratic	2.89	1.37-6.09	**0.0366**
Septic shock	ICU	2028	235	11.6	Quadratic	7.71	3.33-17.89	**<0.001**
Septic shock	0–1 Dysfunctions	5448	211	3.9	Lineal	1.11	0.97-1.28	0.1385
Septic shock	≥2 Dysfunctions	985	290	29.4	Quadratic	2.46	1.42-4.27	**0.0477**

CI: confidence Interval; ED: Emergency Department; ICU: Intensive Care Unit; INR: International Normalized Ratio; OR: Odd Ratio. **Bold** values indicate statistically significant differences ($p \leq 0.05$).

Supplemental Figure S1. Flowchart of Patient Selection and Study Population

Sepsis code activations
Hospital Universitario Son Llàtzer
January 2006 – December 2022

↓

Adult patients (≥18 years) with sepsis or septic shock according to modified Sepsis-2 criteria
N= 14,545

↓

Patients assessed for eligibility
n= 14,276

↓

Exclusions
• Missing INR at sepsis diagnosis (n=6,950)
• Missing platelet count at sepsis diagnosis (n=6,941)

↓

Final study cohort
N = 6,433 patients

↓

Outcome analysis
In-hospital mortality

↓

Sepsis-Induced Coagulopathy (SIC) subgroup
N = 1,421

↓

SIC phenotypes
• Elevated INR only (n=322)
•Thrombocytopenia only (n=284)
• Both altered (DIC pattern) (n=244)
• Mild/subthreshold alterations (n=571)

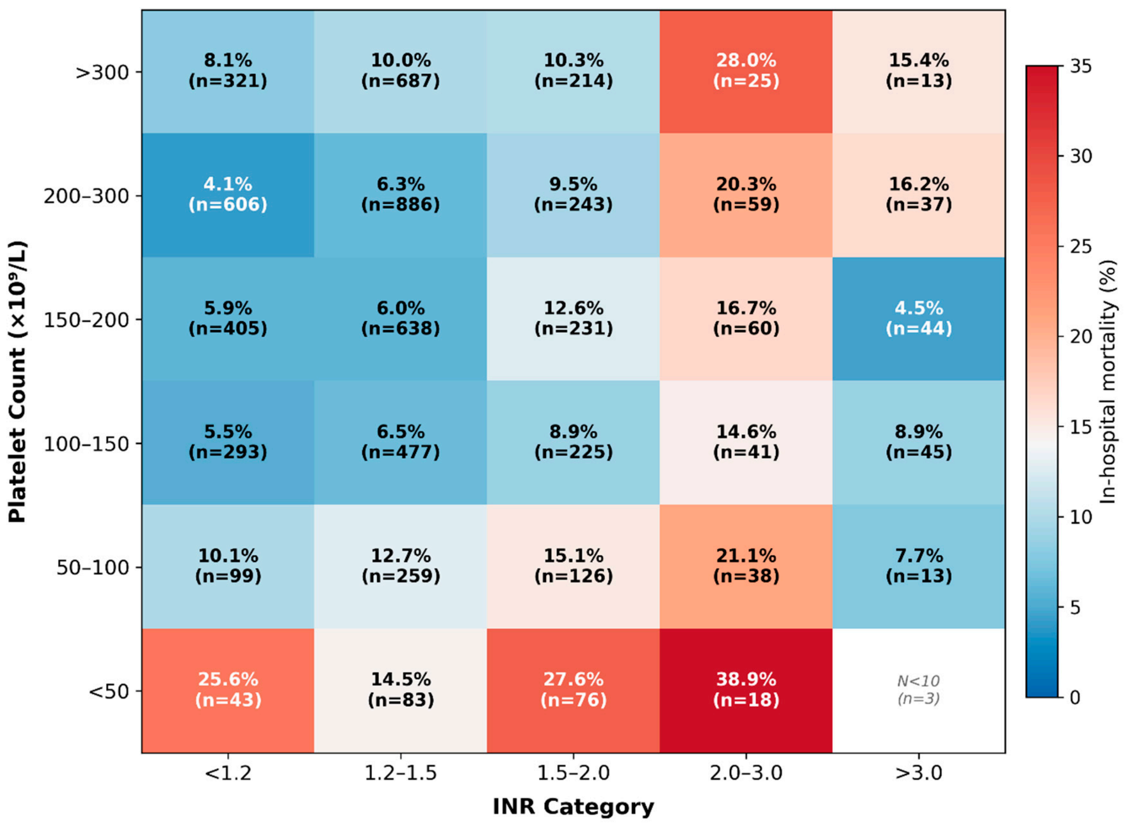

Supplemental Figure S2. Joint in-hospital mortality (%) by INR × platelet count category. Full cohort (N=6,308). Columns: INR categories (<1.2, 1.2–1.5, 1.5–2.0, 2.0–3.0, >3.0). Rows: platelet count categories (<50, 50–100, 100–150, 150–200, 200–300, >300 ×10^9/L). Colour scale: 0–35% mortality. Cells with N<10 shown in grey with italicised annotation. Colour intensity reflects mortality rate (darker = higher risk). Cells with N<10 are excluded. This figure provides contextual visualisation of the joint risk surface and supports the interpretation that INR strata dominate the mortality gradient regardless of platelet count level.

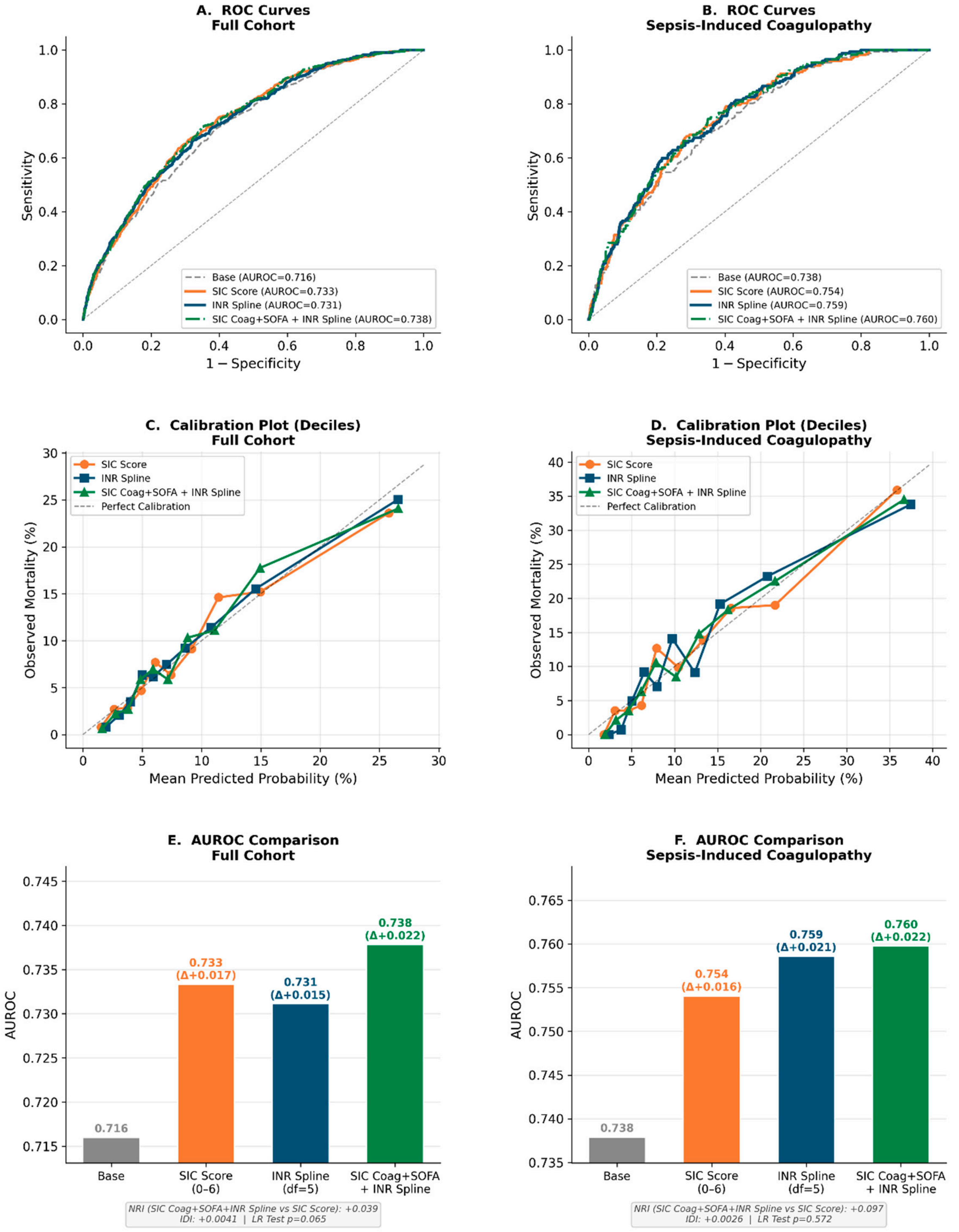

Supplementary Figure S3. Comparison of the INR cubic B-spline model with the SIC score for predicting in-hospital mortality. Upper row: full cohort (N=6,308, mortality 8.8%). Lower row: SIC subgroup (N=1,421, mortality 12.1%). **Panel A/D:** ROC curves for the base model (age + organ dysfunctions, grey dashed), SIC score continuous 0–6 (orange), INR cubic B-spline df=5 (blue), and a modified model replacing the SIC score's dichotomised INR component with the continuous INR spline while retaining the platelet and SOFA components (SIC coag+SOFA + INR spline, green). **Panel B/E:** calibration plots by predicted probability decile; each point represents the mean predicted probability (x-axis) versus observed mortality (y-axis) within each decile; the dashed diagonal represents perfect

calibration. **Panel C/F:** AUROC comparison across the four models; values above bars indicate AUROC and increment over the base model (Δ); inset shows continuous NRI, IDI, and likelihood-ratio test p-value for the modified vs original SIC score. All logistic regression models adjusted for age and non-coagulatory organ dysfunction count (Disf_sinCoag). INR winsorised at the 1st–99th percentile prior to spline fitting.

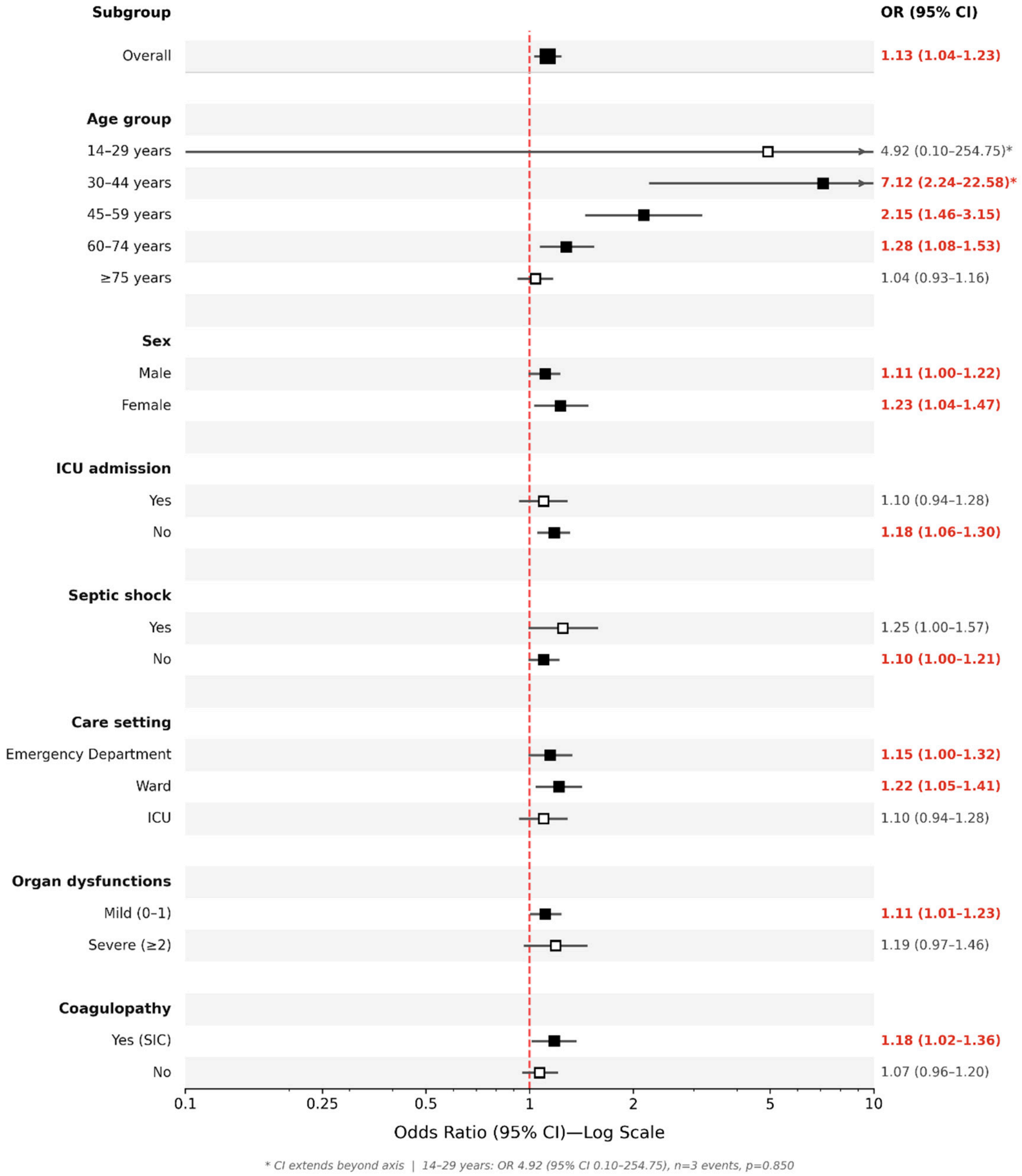

Supplemental Figure S4. Subgroup analysis of INR as prognostic marker for in-hospital mortality. Forest plot displaying odds ratios (OR) and 95% confidence intervals for the association between INR and in-hospital mortality across clinical subgroups including age (14-29, 30-44, 45-59, 60-74, and ≥75 years old), sex (male/female), ICU admission (yes/no), septic shock (yes/no), care settings (emergency department, hospital ward, ICU), clinical severity as number of dysfunctions (low as 0-1 dysfunctions, severe as 2+ dysfunctions), and coagulopathy status (yes/no). Models were adjusted for relevant covariates within each subgroup.
